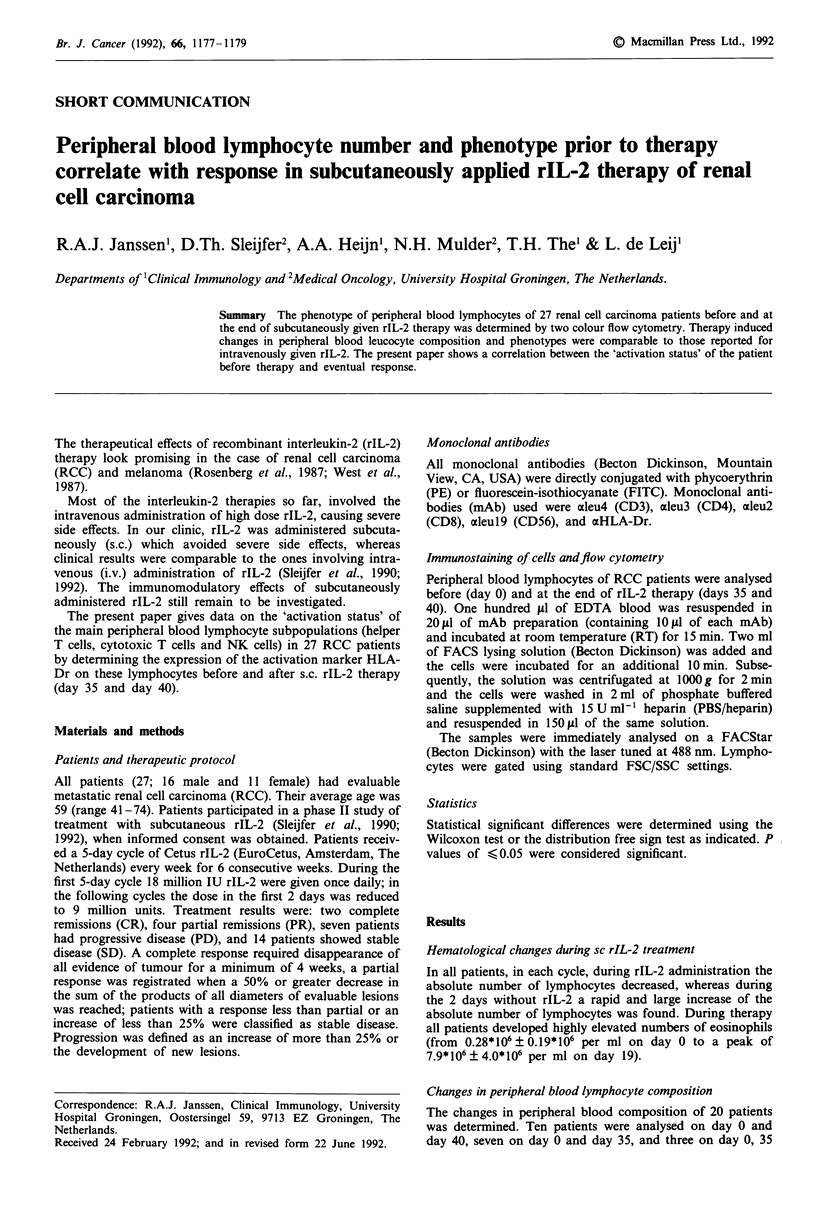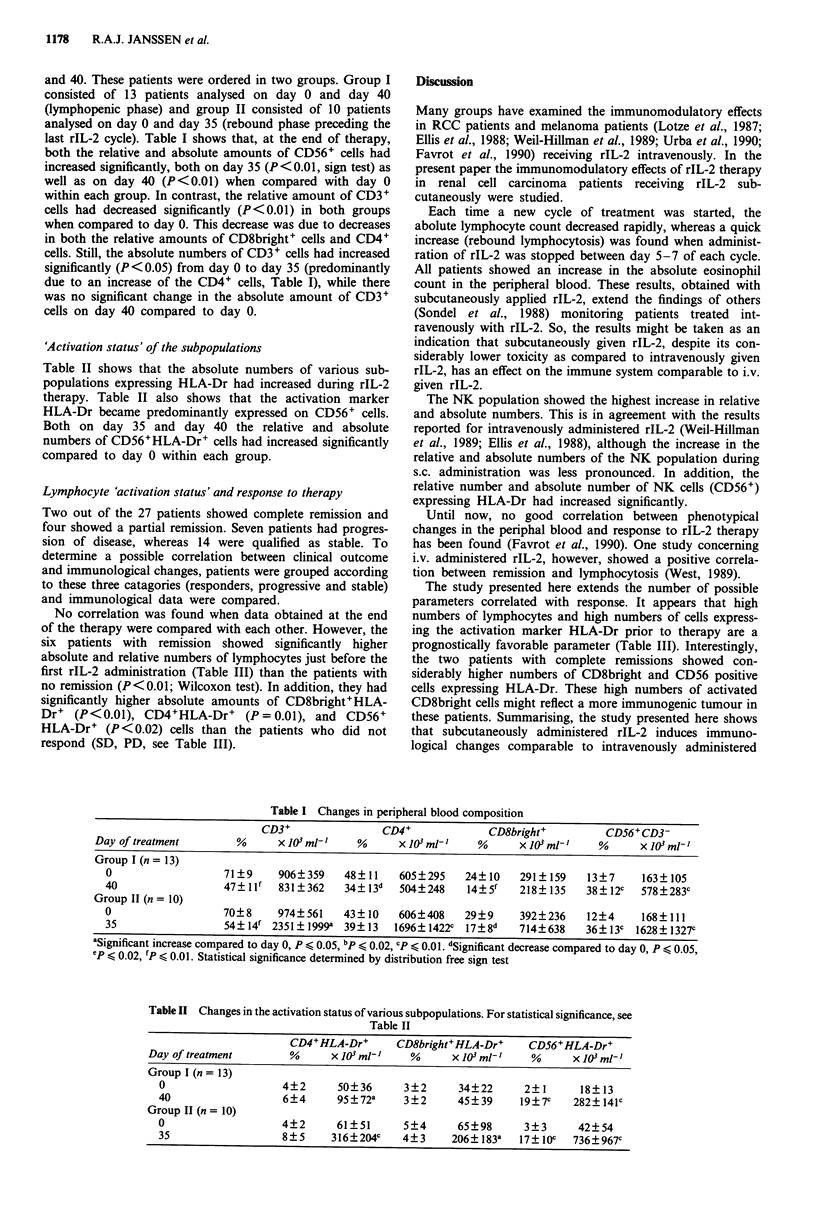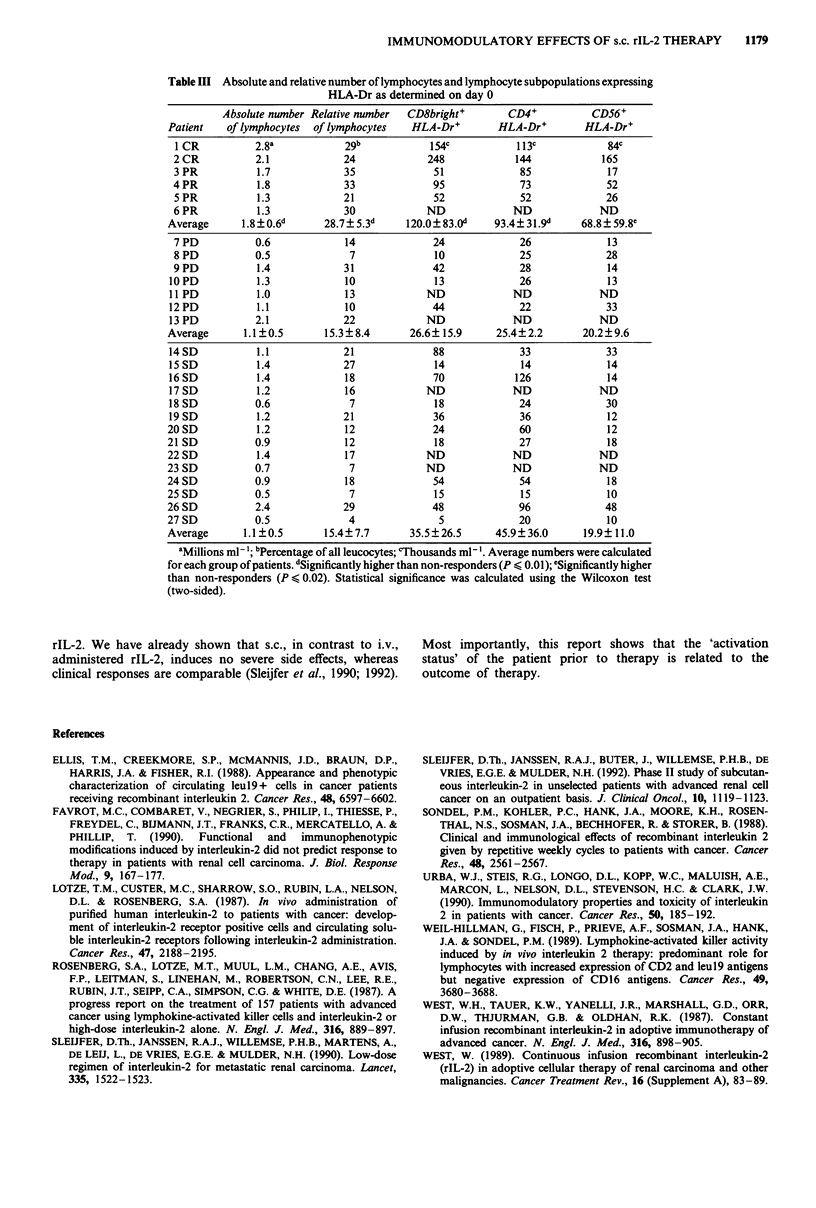# Peripheral blood lymphocyte number and phenotype prior to therapy correlate with response in subcutaneously applied rIL-2 therapy of renal cell carcinoma.

**DOI:** 10.1038/bjc.1992.431

**Published:** 1992-12

**Authors:** R. A. Janssen, D. T. Sleijfer, A. A. Heijn, N. H. Mulder, T. H. The, L. de Leij

**Affiliations:** Department of Clinical Immunology, University Hospital Groningen, The Netherlands.

## Abstract

The phenotype of peripheral blood lymphocytes of 27 renal cell carcinoma patients before and at the end of subcutaneously given rIL-2 therapy was determined by two colour flow cytometry. Therapy induced changes in peripheral blood leucocyte composition and phenotypes were comparable to those reported for intravenously given rIL-2. The present paper shows a correlation between the 'activation status' of the patient before therapy and eventual response.


Br. J. Cancer (1992), 66, 1177–1179 © Macmillan Press Ltd., 1992

SHORT COMMUNICATION

### Peripheral blood lymphocyte number and phenotype prior to therapy correlate with response in subcutaneously applied rIL-2 therapy of renal cell carcinoma

R.A.J. Janssen[1], D.Th. Sleijfer[2], A.A. Heijn[1], N.H. Mulder[2], T.H. The[1] & L. de Leij[1]

*Departments of [1]Clinical Immunology and [2]Medical Oncology, University Hospital Groningen, The Netherlands.*

**Summary** The phenotype of peripheral blood lymphocytes of 27 renal cell carcinoma patients before and at the end of subcutaneously given rIL-2 therapy was determined by two colour flow cytometry. Therapy induced changes in peripheral blood leucocyte composition and phenotypes were comparable to those reported for intravenously given rIL-2. The present paper shows a correlation between the 'activation status' of the patient before therapy and eventual response.

The therapeutical effects of recombinant interleukin-2 (rIL-2) therapy look promising in the case of renal cell carcinoma (RCC) and melanoma (Rosenberg *et al.*, 1987; West *et al.*, 1987).

Most of the interleukin-2 therapies so far, involved the intravenous administration of high dose rIL-2, causing severe side effects. In our clinic, rIL-2 was administered subcutaneously (s.c.) which avoided severe side effects, whereas clinical results were comparable to the ones involving intravenous (i.v.) administration of rIL-2 (Sleijfer *et al.*, 1990; 1992). The immunomodulatory effects of subcutaneously administered rIL-2 still remain to be investigated.

The present paper gives data on the 'activation status' of the main peripheral blood lymphocyte subpopulations (helper T cells, cytotoxic T cells and NK cells) in 27 RCC patients by determining the expression of the activation marker HLA-Dr on these lymphocytes before and after s.c. rIL-2 therapy (day 35 and day 40).

### Materials and methods

### *Patients and therapeutic protocol*

All patients (27; 16 male and 11 female) had evaluable metastatic renal cell carcinoma (RCC). Their average age was 59 (range 41–74). Patients participated in a phase II study of treatment with subcutaneous rIL-2 (Sleijfer *et al.*, 1990; 1992), when informed consent was obtained. Patients received a 5-day cycle of Cetus rIL-2 (EuroCetus, Amsterdam, The Netherlands) every week for 6 consecutive weeks. During the first 5-day cycle 18 million IU rIL-2 were given once daily; in the following cycles the dose in the first 2 days was reduced to 9 million units. Treatment results were: two complete remissions (CR), four partial remissions (PR), seven patients had progressive disease (PD), and 14 patients showed stable disease (SD). A complete response required disappearance of all evidence of tumour for a minimum of 4 weeks, a partial response was registrated when a 50% or greater decrease in the sum of the products of all diameters of evaluable lesions was reached; patients with a response less than partial or an increase of less than 25% were classified as stable disease. Progression was defined as an increase of more than 25% or the development of new lesions.

Correspondence: R.A.J. Janssen, Clinical Immunology, University Hospital Groningen, Oostersingel 59, 9713 EZ Groningen, The Netherlands.
Received 24 February 1992; and in revised form 22 June 1992.

### *Monoclonal antibodies*

All monoclonal antibodies (Becton Dickinson, Mountain View, CA, USA) were directly conjugated with phycoerythrin (PE) or fluorescein-isothiocyanate (FITC). Monoclonal antibodies (mAb) used were αleu4 (CD3), αleu3 (CD4), αleu2 (CD8), αleu19 (CD56), and αHLA-Dr.

### *Immunostaining of cells and flow cytometry*

Peripheral blood lymphocytes of RCC patients were analysed before (day 0) and at the end of rIL-2 therapy (days 35 and 40). One hundred μl of EDTA blood was resuspended in 20 μl of mAb preparation (containing 10 μl of each mAb) and incubated at room temperature (RT) for 15 min. Two ml of FACS lysing solution (Becton Dickinson) was added and the cells were incubated for an additional 10 min. Subsequently, the solution was centrifugated at 1000 *g* for 2 min and the cells were washed in 2 ml of phosphate buffered saline supplemented with 15 U ml$^{-1}$ heparin (PBS/heparin) and resuspended in 150 μl of the same solution.

The samples were immediately analysed on a FACStar (Becton Dickinson) with the laser tuned at 488 nm. Lymphocytes were gated using standard FSC/SSC settings.

### *Statistics*

Statistical significant differences were determined using the Wilcoxon test or the distribution free sign test as indicated. *P* values of $\leqslant 0.05$ were considered significant.

### Results

### *Hematological changes during sc rIL-2 treatment*

In all patients, in each cycle, during rIL-2 administration the absolute number of lymphocytes decreased, whereas during the 2 days without rIL-2 a rapid and large increase of the absolute number of lymphocytes was found. During therapy all patients developed highly elevated numbers of eosinophils (from $0.28*10^6 \pm 0.19*10^6$ per ml on day 0 to a peak of $7.9*10^6 \pm 4.0*10^6$ per ml on day 19).

### *Changes in peripheral blood lymphocyte composition*

The changes in peripheral blood composition of 20 patients was determined. Ten patients were analysed on day 0 and day 40, seven on day 0 and day 35, and three on day 0, 35

and 40. These patients were ordered in two groups. Group I consisted of 13 patients analysed on day 0 and day 40 (lymphopenic phase) and group II consisted of 10 patients analysed on day 0 and day 35 (rebound phase preceding the last rIL-2 cycle). Table I shows that, at the end of therapy, both the relative and absolute amounts of $CD56^+$ cells had increased significantly, both on day 35 ($P<0.01$, sign test) as well as on day 40 ($P<0.01$) when compared with day 0 within each group. In contrast, the relative amount of $CD3^+$ cells had decreased significantly ($P<0.01$) in both groups when compared to day 0. This decrease was due to decreases in both the relative amounts of $CD8bright^+$ cells and $CD4^+$ cells. Still, the absolute numbers of $CD3^+$ cells had increased significantly ($P<0.05$) from day 0 to day 35 (predominantly due to an increase of the $CD4^+$ cells, Table I), while there was no significant change in the absolute amount of $CD3^+$ cells on day 40 compared to day 0.

*'Activation status' of the subpopulations*

Table II shows that the absolute numbers of various subpopulations expressing HLA-Dr had increased during rIL-2 therapy. Table II also shows that the activation marker HLA-Dr became predominantly expressed on $CD56^+$ cells. Both on day 35 and day 40 the relative and absolute numbers of $CD56^+HLA\text{-}Dr^+$ cells had increased significantly compared to day 0 within each group.

*Lymphocyte 'activation status' and response to therapy*

Two out of the 27 patients showed complete remission and four showed a partial remission. Seven patients had progression of disease, whereas 14 were qualified as stable. To determine a possible correlation between clinical outcome and immunological changes, patients were grouped according to these three catagories (responders, progressive and stable) and immunological data were compared.

No correlation was found when data obtained at the end of the therapy were compared with each other. However, the six patients with remission showed significantly higher absolute and relative numbers of lymphocytes just before the first rIL-2 administration (Table III) than the patients with no remission ($P<0.01$; Wilcoxon test). In addition, they had significantly higher absolute amounts of $CD8bright^+HLA\text{-}Dr^+$ ($P<0.01$), $CD4^+HLA\text{-}Dr^+$ ($P=0.01$), and $CD56^+$ $HLA\text{-}Dr^+$ ($P<0.02$) cells than the patients who did not respond (SD, PD, see Table III).

### Discussion

Many groups have examined the immunomodulatory effects in RCC patients and melanoma patients (Lotze et al., 1987; Ellis et al., 1988; Weil-Hillman et al., 1989; Urba et al., 1990; Favrot et al., 1990) receiving rIL-2 intravenously. In the present paper the immunomodulatory effects of rIL-2 therapy in renal cell carcinoma patients receiving rIL-2 subcutaneously were studied.

Each time a new cycle of treatment was started, the abolute lymphocyte count decreased rapidly, whereas a quick increase (rebound lymphocytosis) was found when administration of rIL-2 was stopped between day 5–7 of each cycle. All patients showed an increase in the absolute eosinophil count in the peripheral blood. These results, obtained with subcutaneously applied rIL-2, extend the findings of others (Sondel et al., 1988) monitoring patients treated intravenously with rIL-2. So, the results might be taken as an indication that subcutaneously given rIL-2, despite its considerably lower toxicity as compared to intravenously given rIL-2, has an effect on the immune system comparable to i.v. given rIL-2.

The NK population showed the highest increase in relative and absolute numbers. This is in agreement with the results reported for intravenously administered rIL-2 (Weil-Hillman et al., 1989; Ellis et al., 1988), although the increase in the relative and absolute numbers of the NK population during s.c. administration was less pronounced. In addition, the relative number and absolute number of NK cells ($CD56^+$) expressing HLA-Dr had increased significantly.

Until now, no good correlation between phenotypical changes in the periphal blood and response to rIL-2 therapy has been found (Favrot et al., 1990). One study concerning i.v. administered rIL-2, however, showed a positive correlation between remission and lymphocytosis (West, 1989).

The study presented here extends the number of possible parameters correlated with response. It appears that high numbers of lymphocytes and high numbers of cells expressing the activation marker HLA-Dr prior to therapy are a prognostically favorable parameter (Table III). Interestingly, the two patients with complete remissions showed considerably higher numbers of CD8bright and CD56 positive cells expressing HLA-Dr. These high numbers of activated CD8bright cells might reflect a more immunogenic tumour in these patients. Summarising, the study presented here shows that subcutaneously administered rIL-2 induces immunological changes comparable to intravenously administered

**Table I** Changes in peripheral blood composition

| Day of treatment | $CD3^+$ % | $CD3^+$ $\times 10^3\,ml^{-1}$ | $CD4^+$ % | $CD4^+$ $\times 10^3\,ml^{-1}$ | $CD8bright^+$ % | $CD8bright^+$ $\times 10^3\,ml^{-1}$ | $CD56^+CD3^-$ % | $CD56^+CD3^-$ $\times 10^3\,ml^{-1}$ |
|---|---|---|---|---|---|---|---|---|
| Group I ($n = 13$) | | | | | | | | |
| 0 | 71±9 | 906±359 | 48±11 | 605±295 | 24±10 | 291±159 | 13±7 | 163±105 |
| 40 | 47±11[f] | 831±362 | 34±13[d] | 504±248 | 14±5[f] | 218±135 | 38±12[c] | 578±283[c] |
| Group II ($n = 10$) | | | | | | | | |
| 0 | 70±8 | 974±561 | 43±10 | 606±408 | 29±9 | 392±236 | 12±4 | 168±111 |
| 35 | 54±14[f] | 2351±1999[a] | 39±13 | 1696±1422[c] | 17±8[d] | 714±638 | 36±13[c] | 1628±1327[c] |

[a]Significant increase compared to day 0, $P \leqslant 0.05$, [b]$P \leqslant 0.02$, [c]$P \leqslant 0.01$. [d]Significant decrease compared to day 0, $P \leqslant 0.05$, [e]$P \leqslant 0.02$, [f]$P \leqslant 0.01$. Statistical significance determined by distribution free sign test

**Table II** Changes in the activation status of various subpopulations. For statistical significance, see Table II

| Day of treatment | $CD4^+HLA\text{-}Dr^+$ % | $CD4^+HLA\text{-}Dr^+$ $\times 10^3\,ml^{-1}$ | $CD8bright^+HLA\text{-}Dr^+$ % | $CD8bright^+HLA\text{-}Dr^+$ $\times 10^3\,ml^{-1}$ | $CD56^+HLA\text{-}Dr^+$ % | $CD56^+HLA\text{-}Dr^+$ $\times 10^3\,ml^{-1}$ |
|---|---|---|---|---|---|---|
| Group I ($n = 13$) | | | | | | |
| 0 | 4±2 | 50±36 | 3±2 | 34±22 | 2±1 | 18±13 |
| 40 | 6±4 | 95±72[a] | 3±2 | 45±39 | 19±7[c] | 282±141[c] |
| Group II ($n = 10$) | | | | | | |
| 0 | 4±2 | 61±51 | 5±4 | 65±98 | 3±3 | 42±54 |
| 35 | 8±5 | 316±204[c] | 4±3 | 206±183[a] | 17±10[c] | 736±967[c] |

**Table III** Absolute and relative number of lymphocytes and lymphocyte subpopulations expressing HLA-Dr as determined on day 0

| *Patient* | *Absolute number of lymphocytes* | *Relative number of lymphocytes* | *CD8bright⁺ HLA-Dr⁺* | *CD4⁺ HLA-Dr⁺* | *CD56⁺ HLA-Dr⁺* |
|---|---|---|---|---|---|
| 1 CR | 2.8[a] | 29[b] | 154[c] | 113[c] | 84[c] |
| 2 CR | 2.1 | 24 | 248 | 144 | 165 |
| 3 PR | 1.7 | 35 | 51 | 85 | 17 |
| 4 PR | 1.8 | 33 | 95 | 73 | 52 |
| 5 PR | 1.3 | 21 | 52 | 52 | 26 |
| 6 PR | 1.3 | 30 | ND | ND | ND |
| Average | 1.8±0.6[d] | 28.7±5.3[d] | 120.0±83.0[d] | 93.4±31.9[d] | 68.8±59.8[e] |
| 7 PD | 0.6 | 14 | 24 | 26 | 13 |
| 8 PD | 0.5 | 7 | 10 | 25 | 28 |
| 9 PD | 1.4 | 31 | 42 | 28 | 14 |
| 10 PD | 1.3 | 10 | 13 | 26 | 13 |
| 11 PD | 1.0 | 13 | ND | ND | ND |
| 12 PD | 1.1 | 10 | 44 | 22 | 33 |
| 13 PD | 2.1 | 22 | ND | ND | ND |
| Average | 1.1±0.5 | 15.3±8.4 | 26.6±15.9 | 25.4±2.2 | 20.2±9.6 |
| 14 SD | 1.1 | 21 | 88 | 33 | 33 |
| 15 SD | 1.4 | 27 | 14 | 14 | 14 |
| 16 SD | 1.4 | 18 | 70 | 126 | 14 |
| 17 SD | 1.2 | 16 | ND | ND | ND |
| 18 SD | 0.6 | 7 | 18 | 24 | 30 |
| 19 SD | 1.2 | 21 | 36 | 36 | 12 |
| 20 SD | 1.2 | 12 | 24 | 60 | 12 |
| 21 SD | 0.9 | 12 | 18 | 27 | 18 |
| 22 SD | 1.4 | 17 | ND | ND | ND |
| 23 SD | 0.7 | 7 | ND | ND | ND |
| 24 SD | 0.9 | 18 | 54 | 54 | 18 |
| 25 SD | 0.5 | 7 | 15 | 15 | 10 |
| 26 SD | 2.4 | 29 | 48 | 96 | 48 |
| 27 SD | 0.5 | 4 | 5 | 20 | 10 |
| Average | 1.1±0.5 | 15.4±7.7 | 35.5±26.5 | 45.9±36.0 | 19.9±11.0 |

[a]Millions $ml^{-1}$; [b]Percentage of all leucocytes; [c]Thousands $ml^{-1}$. Average numbers were calculated for each group of patients. [d]Significantly higher than non-responders ($P \leqslant 0.01$); [e]Significantly higher than non-responders ($P \leqslant 0.02$). Statistical significance was calculated using the Wilcoxon test (two-sided).

rIL-2. We have already shown that s.c., in contrast to i.v., administered rIL-2, induces no severe side effects, whereas clinical responses are comparable (Sleijfer *et al.*, 1990; 1992). Most importantly, this report shows that the 'activation status' of the patient prior to therapy is related to the outcome of therapy.

### References

ELLIS, T.M., CREEKMORE, S.P., MCMANNIS, J.D., BRAUN, D.P., HARRIS, J.A. & FISHER, R.I. (1988). Appearance and phenotypic characterization of circulating leu19+ cells in cancer patients receiving recombinant interleukin 2. *Cancer Res.*, **48**, 6597–6602.

FAVROT, M.C., COMBARET, V., NEGRIER, S., PHILIP, I., THIESSE, P., FREYDEL, C., BIJMANN, J.T., FRANKS, C.R., MERCATELLO, A. & PHILLIP, T. (1990). Functional and immunophenotypic modifications induced by interleukin-2 did not predict response to therapy in patients with renal cell carcinoma. *J. Biol. Response Mod.*, **9**, 167–177.

LOTZE, T.M., CUSTER, M.C., SHARROW, S.O., RUBIN, L.A., NELSON, D.L. & ROSENBERG, S.A. (1987). *In vivo* administration of purified human interleukin-2 to patients with cancer: development of interleukin-2 receptor positive cells and circulating soluble interleukin-2 receptors following interleukin-2 administration. *Cancer Res.*, **47**, 2188–2195.

ROSENBERG, S.A., LOTZE, M.T., MUUL, L.M., CHANG, A.E., AVIS, F.P., LEITMAN, S., LINEHAN, M., ROBERTSON, C.N., LEE, R.E., RUBIN, J.T., SEIPP, C.A., SIMPSON, C.G. & WHITE, D.E. (1987). A progress report on the treatment of 157 patients with advanced cancer using lymphokine-activated killer cells and interleukin-2 or high-dose interleukin-2 alone. *N. Engl. J. Med.*, **316**, 889–897.

SLEIJFER, D.Th., JANSSEN, R.A.J., WILLEMSE, P.H.B., MARTENS, A., DE LEIJ, L., DE VRIES, E.G.E. & MULDER, N.H. (1990). Low-dose regimen of interleukin-2 for metastatic renal carcinoma. *Lancet*, **335**, 1522–1523.

SLEIJFER, D.Th., JANSSEN, R.A.J., BUTER, J., WILLEMSE, P.H.B., DE VRIES, E.G.E. & MULDER, N.H. (1992). Phase II study of subcutaneous interleukin-2 in unselected patients with advanced renal cell cancer on an outpatient basis. *J. Clinical Oncol.*, **10**, 1119–1123.

SONDEL, P.M., KOHLER, P.C., HANK, J.A., MOORE, K.H., ROSENTHAL, N.S., SOSMAN, J.A., BECHHOFER, R. & STORER, B. (1988). Clinical and immunological effects of recombinant interleukin 2 given by repetitive weekly cycles to patients with cancer. *Cancer Res.*, **48**, 2561–2567.

URBA, W.J., STEIS, R.G., LONGO, D.L., KOPP, W.C., MALUISH, A.E., MARCON, L., NELSON, D.L., STEVENSON, H.C. & CLARK, J.W. (1990). Immunomodulatory properties and toxicity of interleukin 2 in patients with cancer. *Cancer Res.*, **50**, 185–192.

WEIL-HILLMAN, G., FISCH, P., PRIEVE, A.F., SOSMAN, J.A., HANK, J.A. & SONDEL, P.M. (1989). Lymphokine-activated killer activity induced by *in vivo* interleukin 2 therapy: predominant role for lymphocytes with increased expression of CD2 and leu19 antigens but negative expression of CD16 antigens. *Cancer Res.*, **49**, 3680–3688.

WEST, W.H., TAUER, K.W., YANELLI, J.R., MARSHALL, G.D., ORR, D.W., THJURMAN, G.B. & OLDHAN, R.K. (1987). Constant infusion recombinant interleukin-2 in adoptive immunotherapy of advanced cancer. *N. Engl. J. Med.*, **316**, 898–905.

WEST, W. (1989). Continuous infusion recombinant interleukin-2 (rIL-2) in adoptive cellular therapy of renal carcinoma and other malignancies. *Cancer Treatment Rev.*, **16** (Supplement A), 83–89.